# The Search for Environmental Justice: The Story of North Birmingham

**DOI:** 10.3390/ijerph16122117

**Published:** 2019-06-14

**Authors:** Shauntice Allen, Michelle V. Fanucchi, Lisa C. McCormick, Kristina M. Zierold

**Affiliations:** Department of Environmental Health Sciences, School of Public Health, University of Alabama at Birmingham, 1665 University Blvd., Birmingham, AL 35294-0022, USA; fanucchi@uab.edu (M.V.F.); lmccormick@uab.edu (L.C.M.); kzierold@uab.edu (K.M.Z.)

**Keywords:** environmental justice, pollution, Superfund, industrial pollution, contaminated sites, disadvantaged groups

## Abstract

Environmental justice is a rising social movement throughout the world. Research is beginning to define the movement and address the disparities that exist among communities exposed to pollution. North Birmingham, a community made up of six neighborhoods in Jefferson County, Alabama, in the United States, is a story of environmental injustice. Heavy industry, including the 35th Avenue Superfund Site, has caused significant environmental pollution over time, leaving residents concerned that their health and well-being are at risk from continued exposure. For years, pollution has impacted the community, and residents have fought and challenged industry and government. The United States (U.S.) Environmental Protection Agency (EPA), the U.S. Agency for Toxic Substances and Disease Registry (ATSDR), and the Jefferson County Department of Health (JCDH) in Alabama have historically played a role in working with the community regarding their health concerns. In this manuscript, we describe a city entrenched in environmental injustice. We provide the history of the community, the responsible parties named for the contamination, the government’s involvement, and the community’s response to this injustice. Through this manuscript, we offer insight into a global concern that challenges local communities on a daily basis.

## 1. Introduction

Environmental justice is a rising social movement throughout the world. While limited scientific research has been conducted worldwide, researchers are beginning to confirm the need for more studies addressing environmental justice. The United States Environmental Protection Agency (EPA) defines environmental justice as “the fair treatment and meaningful involvement of all people regardless of race, color, national origin, or income with respect to the development, implementation, and enforcement of environmental laws, regulations, and policies” [1]. “Fair treatment” means that no group, due to policy or economic disempowerment, bears a disproportionate share of the negative human health or environmental impacts of pollution or the environmental consequences resulting from industrial, municipal, and commercial operations or the execution of federal, state, local, and tribal programs and policies. “Meaningful involvement” allows people to actively participate in decisions about activities that affect their environment and/or health.

Beginning in the early 1970s, a substantial body of literature was developed that documented the existence of environmental inequalities in the United States [2,3]. These early findings were followed by a series of studies focused on the location of hazardous waste sites, beginning with a study conducted by the United States (U.S.) General Accounting Office in 1983. This study documented that African American communities in the southern United States were hosts to a disproportionately high number of waste sites [4]. This regional study was followed in 1987 by the United Church of Christ (UCC) Commission on Racial Justice’s groundbreaking national study titled *Toxic Waste and Race in the United States* [5]. This study documented the unequal and discriminatory siting of toxic waste facilities across the United States. These data collection and reporting activities brought a sharper focus to the intersection of civil rights and environmental rights. The UCC study concluded that race was the most important variable in predicting where these sites would be located, more powerful than poverty, land values, and home ownership. Recent studies have also confirmed that race is the most powerful predictor in determining where toxic facilities are located [6,7].

In February 1994, Executive Order 12898, known as “Federal Actions to Address Environmental Justice in Minority Populations and Low-Income Populations”, was promulgated. Order 12898 directed U.S. federal agencies, such as the Environmental Protection Agency (EPA), Health and Human Services (HHS), Housing and Urban Development (HUD), and the Department of Transportation (DOT) to address environmental justice through programming, policies, and activities [8]. The order specified that an interagency working group be formed to provide guidance as each agency developed its plan to address environmental justice and assist in the coordination of research regarding environmental justice. Since the original order, federal agencies have continued forward in their work around environmental justice.

Since the aforementioned groundbreaking studies, more research throughout the world has been conducted surrounding, race, poverty, and pollution [9,10,11,12,13,14]. In a recent study assessing air pollution and noise exposure in Belgium, Verbeek found that lower income neighborhoods with more unemployment, more people of foreign origin, more rental housing, and higher residential mobility were more exposed to air pollution compared to other residents [12]. Environmental justice issues related to drinking water have been reported in communities such as the First Nations communities in Ontario, Canada, and in Flint, Michigan [9,10]. In countries outside the U.S., there has been a lack of research on environmental justice, particularly as it relates to community health. In a review article by Passetto et al., the authors highlighted that outside of the U.S. and the World Health Organization (WHO) European Region, only 11 studies focused on environmental justice between 2010 and 2017 [14].

Minority and poor communities are often on the front lines of the environmental assault on their neighborhoods. Unfortunately, the burden of proof is often left up to communities, where racism and poverty constrain residents from fully participating in the decisions that impact their lives. The purpose of this paper is to examine the community of North Birmingham as it seeks environmental justice. We will describe the history of the community, industry’s responsibility for contamination, government involvement, legal battles, and the community’s response to this injustice. 

## 2. The North Birmingham Community

**Figure 1:**
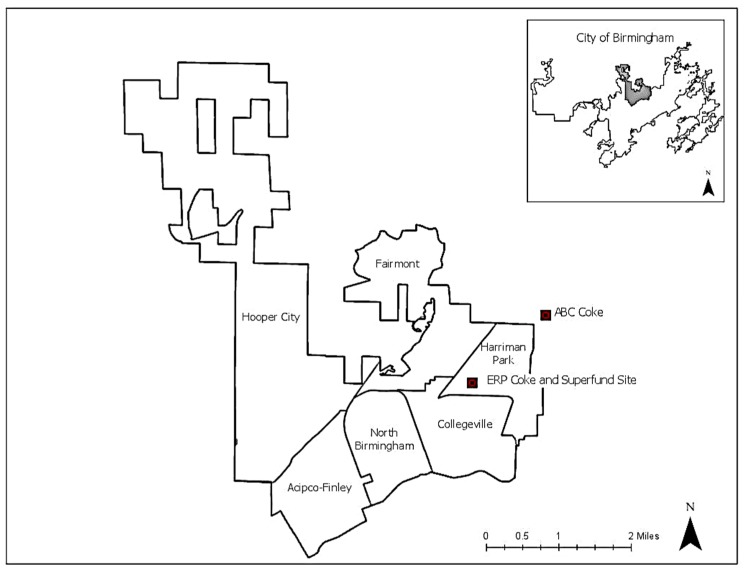
North Birmingham neighborhoods and coke plants.

North Birmingham was originally incorporated as the city of North Birmingham in 1902 and was later annexed into the City of Birmingham in 1910 [15]. In the early 1900s, North Birmingham had approximately 3500 people. Many of the neighborhoods in North Birmingham were initially developed by the companies that employed residents, creating a working class cultural and architectural character that is still visible today [15,16,17]. The current 35th Ave Superfund Site was once home to parks, a country club, and a nine-hole golf course. The communities of North Birmingham played a significant role in shaping the cultural and social identity of greater Birmingham through its civic leaders and involvement in the Civil Rights movement, including the Children’s March (personal communication from North Birmingham Community Coalition Members). A defining feature of civic life and organization in Birmingham is the Citizens Advisory Board (CAB). Formed in 1974 to improve communication between residents and city leaders, the CAB is a structured network of 99 neighborhoods aggregated into 23 larger units called “communities”. Each neighborhood has a neighborhood association that serves as the primary conduit for communicating specific issues, problems, and opportunities to the city government [18]. The North Birmingham community includes the neighborhoods of Acipco Finley, Collegeville, Fairmont, Harriman Park, Hooper City, and North Birmingham (see Figure 1) [16]. 

**Figure 2:**
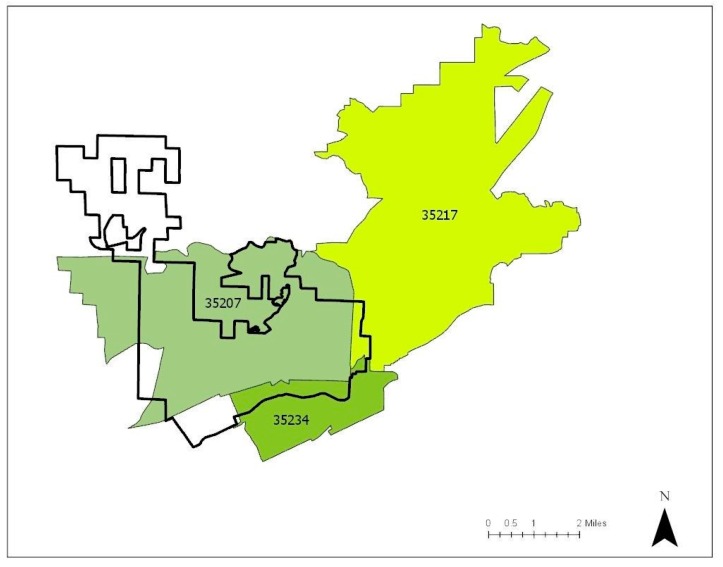
Zip codes impacted by heavy industry and the 35th Avenue Superfund Site.

North Birmingham and adjacent neighborhoods affected by heavy industrial pollution and the 35th Avenue Superfund Site are located in the zip codes of 35207, 35217, and 35234 (see Figure 2). In addition to the Superfund Site, the EPA’s toxic release inventory (TRI) in 2019 showed that the area of North Birmingham contains the top four industries responsible for chemical releases.

Table 1 presents the demographics of these areas. As shown in the table, the populations in the three zip codes are primarily African American, have lower average annual incomes, have higher percentages of people living below the poverty level, and have populations that are less likely to have health insurance compared to Jefferson County, Alabama [19].

Despite North Birmingham’s significant history, land use and zoning decisions have resulted in isolated neighborhoods with limited amenities (i.e., healthcare, healthy food options, transportation). Birmingham chose to ignore the 1917 Supreme Court ruling *Buchanan v. Warley* that overturned racial zoning ordinances by arguing that threats to peace were imminent and severe if African Americans and whites lived in the same neighborhoods [20]. The need to maintain order overruled the constitutional rights of those involved. Essentially, government policy kept the location racially segregated through zoning ordinances for decades, with racial zoning maps used to guide its commercial and residential planning in the following decades. Heavy industry in this area and the resulting 35th Avenue Superfund Site have caused significant environmental pollution over time, leaving residents concerned that their health and well-being are at risk from continued exposure.

## 3. Description and Location of Industry and the Superfund Site

### 3.1. North Birmingham Coke Plants

North Birmingham has a history of steel production involving facilities such as coke plants. Coke plants are responsible for burning coal to produce coke, which is used in blast furnaces. The production of coke creates solid, airborne, and waterborne waste [21]. Coke oven emissions are classified as known human carcinogens. These emissions are complex mixtures comprised of components such as formaldehyde, carbon monoxide, phenol, arsenic, cadmium, mercury, polycyclic aromatic hydrocarbons (PAHs), and aliphatic aldehydes. More than 60 organic compounds, including 40 PAHs, have been collected in air samples near coke plants. As of 2016, there were 16 operating coke plants in the U.S. [22,23].

Within and adjacent to the North Birmingham community are two coal-fire-powered coke plants, ERP Compliant Coke, LLC, and ABC Coke. The ERP Coke facility, formerly known as Sloss Industries Corporation and later as Walter Coke, has been in operation at its present location since 1920. When ERP purchased the site from Walter Coke in February 2016, it assumed the environmental responsibilities of a 2012 EPA order. Currently, ERP Coke, LLC, produces approximately 460,000 tons of coke annually [23].

ABC Coke is located on the northeast border of the North Birmingham community in the city of Tarrant. Construction of the coke plant began on this site in 1918, which was known as Alabama By-Products Corporation (ABC) by 1920. This site has continuously operated by producing coke and its primary byproducts: Ammonia, benzol, and coal tar. Drummond Company, Inc., merged with ABC in 1985, and the site became the ABC Coke Division of Drummond. Drummond reports that the Tarrant plant currently produces 2150 tons of coke per day and is the largest producer of foundry coke in the U.S. [24].

### 3.2. 35th Avenue Superfund Site

In 2011, after the EPA found toxic contaminants leaching offsite from one of the local coke plants, the EPA utilized its emergency Superfund authority to become involved in the North Birmingham neighborhoods. Created from a tax on the chemical and petroleum industry in response to high-profile toxic waste dumps such as in Love Canal, New York, and the Valley of the Drums in Kentucky, the Superfund program allows the EPA to identify and hold liable potentially responsible parties (i.e., generators or transporters of the hazardous waste on the site, and past or present owners of the site) and establishes a trust fund for cleanup of these sites when no responsible party is identified [25,26]. There are often misconceptions in communities about what the EPA Superfund program is and what it can or cannot do for a community, particularly as it relates to a site being placed on the National Priorities List (NPL) and accessing Superfund resources. Although the EPA Superfund program can immediately begin cleanup (or removal actions) when levels of contaminants are of immediate threat to human health, long-term remediation funds are only available to Superfund sites that are listed on the NPL. In general, a site may be proposed for the NPL in one of three ways: 1) A site assessment results in a hazard ranking score (HRS) of 28.5 or higher, 2) the site is designated by a state or territory as its highest priority for cleanup (each state is allowed only one such designation), or 3) the Agency for Toxic Substance and Disease Registry (ATSDR) issues a health advisory for the site [27,28].

In October 2012, the EPA began its effort around the 35th Avenue Site by seeking access to residential properties. The EPA notified 2049 residential properties that their soil needed to be tested for contamination from semivolatile organic compounds, metals (including arsenic and lead), and polycyclic aromatic hydrocarbons (PAHs), including benzo(a)pyrene, benzo(a)anthrocene, and benzo(b)fluoranthene [29]. These pollutants are emitted from a variety of sources in the area, including coke ovens and smoke stacks. In February 2014, the EPA began removal action cleanup. Over 1900 properties have been tested, with 700 properties being contaminated above Superfund removal management levels. Approximately 400 properties have had their soil removed. On 22 September 2014, the 35th Avenue Site was proposed to the NPL with an HRS score of 50, well above the score needed to be placed on the NPL list [30]. While the 35th Avenue Superfund Site had a score well above the score needed for the NPL, to date, the site has not been listed.

### 3.3. Additional Industry Contributing to Environmental Pollution

While ERP Coke is a potentially responsible party for the contamination found at the 35th Avenue Superfund Site, and its facility encompasses the largest geographical area, the EPA has identified other industrial facilities as potentially responsible parties. These include the Drummond Company (which owns the ABC Coke plant in Tarrant, AL, and was discussed above), the United States Pipe and Foundry Company (also known as U.S. Pipe), the Alabama Gas Company (Alagasco), and the Process Knowledge Corporation (doing business as KMAC Services) [31]. Each potentially responsible party may be responsible under the Superfund for cleanup of the 35th Avenue Superfund Site or costs the EPA incurs when cleaning up the site.

In addition to those named as potentially responsible parties or possible contributors to the contamination found at the 35th Avenue Superfund Site, there are other industrial sites located in North Birmingham that contribute to the overall pollution load in the community. The EPA’s Toxic Release Inventory (TRI) tracks self-reported releases of chemicals to the air, water, and/or land that may pose a threat to the environment or human health. Facilities are required to report to the EPA’s TRI if they manufacture, process, or use certain chemicals past a threshold amount. The facilities in the North Birmingham area with the largest reported releases include the American Cast Iron Pipe Co. and Akzo Nobel Coatings, Inc. In 2012, the American Cast Iron Pipe Co. reported that they released more than 1.4 million pounds of chemicals into the air, water, or land. Akzo Nobel Coatings, Inc., reported releases of more than 47,000 pounds [31].

### 3.4. Industry and Risk

**Figure 3:**
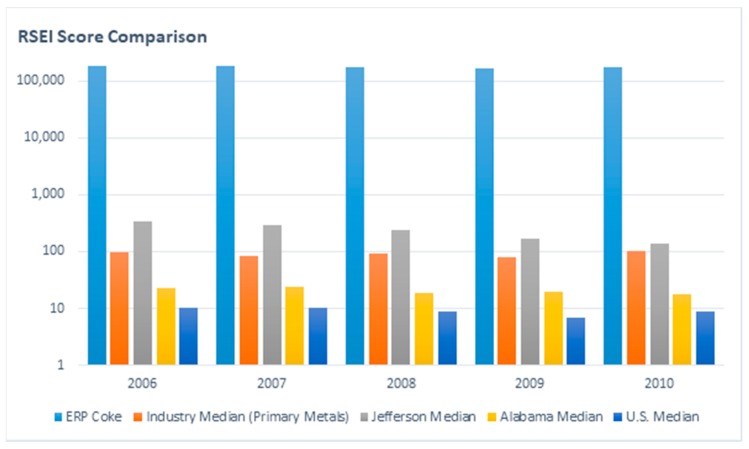
Risk-Screening Environmental Indicators (RSEI) risk scores for ERP Coke and other facilities.

In 2017, there were 32 TRI facilities in Birmingham, which released 1.9 million pounds of chemicals [32]. Out of 56 states/territories nationwide, Alabama ranks 10th based on total releases per square mile. The Risk-Screening Environmental Indicators (RSEI) model is a computer-based screening tool developed by the U.S. EPA that analyzes factors that may result in chronic human health risks [33]. The releases documented in the TRI are facility-reported data. Figure 3 shows that ERP Coke (light blue) has a much greater RSEI risk score compared to the industry median (e.g., other coke plants) (orange) RSEI risk score; the Jefferson County, Alabama, median (gray) RSEI risk score; the state of Alabama median (yellow) RSEI risk score; and the U.S. median (darker blue) RSEI risk score. Throughout the five-year time period reported, ERP Coke was much higher than the other categories. The results in Figure 3 suggest that ERP Coke poses a significantly higher environmental health risk than other facilities.

## 4. Community Health Concerns

A report by the Institute of Medicine [34] concluded that the government, public health officials, and the medical and scientific communities need to place a higher value on the problems and concerns of environmental justice communities. North Birmingham residents have expressed in neighborhood meetings, larger public meetings, and to city leadership their concerns regarding their health and health risks. In 2015, a Title IV civil rights complaint was filed with the EPA against the Jefferson County Department of Health (JCDH) after a request was denied for a hearing on the issuance of Walter Coke’s air permit. The petition requested that the EPA investigate the alleged disparate impacts of air pollution in majority African American neighborhoods near Walter Coke [35].

Community concerns included particulate matter on personal and real property and unpleasant emission odors from the Walter Coke facility that were interfering with sleep and causing irritation of the upper respiratory tract. Other health concerns included sinus headaches and infections and heightened symptoms of chronic obstructive pulmonary disease (COPD), asthma, and cancer. Concerns about contamination of yards and gardens were other expressed complaints. Residents have continuously expressed that a board of health comprised of physicians and a county commissioner did not take the time and opportunity to prevent the myriad health concerns exacerbated by heavy industry emissions. Health professionals and government leadership often internalized the community’s concerns, inhibiting a meaningful relationship from being established.

## 5. Government Response to the Health Concerns

The EPA requested that the ATSDR assess environmental data collected from the Collegeville/Fairmont/Harriman Park neighborhoods in North Birmingham. The evaluation of the data resulted in a 2015 public health assessment (PHA) to determine exposure and whether hazards needed to be reduced or stopped [36]. PHAs are required for all sites listed on the EPA NPL or when concerned individuals petition for an assessment. The PHA in North Birmingham was conducted specifically to determine if exposure to air pollutants was a public health threat to the people living and working in the Collegeville/Fairmont/Harriman Park neighborhoods. 

Samples for the 2015 PHA were collected by the EPA and the county health department. Air samples were collected and analyzed for 102 contaminants in 2005/2006. The EPA collected air samples in 2009 and had them analyzed for 59 contaminants. Again in 2011 and 2012, the EPA collected air samples, but this time analyzed them for 91 contaminants. These air samples were the basis for the PHA. Based on the 2015 PHA regarding exposure to air pollutants, the ATSDR stated that “exposures to particulate matter in North Birmingham air in the past (1999–2012) could have resulted in harmful effects in sensitive individuals but not the general public. Population subgroups that may be more sensitive to the effects of particulate matter exposure include children (under 18 years of age), older adults (over 65 years old), individuals with asthma, chronic obstructive pulmonary disease (COPD), or cardiovascular disease, diabetics, lower socioeconomic status, and those with certain genetic predispositions” [36]. However, it also concluded that “current exposures to particulate matter in North Birmingham air are unlikely to result in harmful effects in individuals.” Concentrations of PM_10_ in North Birmingham decreased from a maximum 24-h average concentration of 136 µg/m^3^ in 1999 to 114 µg/m^3^ in 2005 to 65 µg/m^3^ in 2011 [36]. Likewise, concentrations of PM_2.5_ decreased over the same time period. Table 2 displays results from the North Birmingham monitors for PM_2.5_.

In addition to particulate matter, samples were analyzed for volatile organic compounds, carbonyls, and metals. The ATSDR stated that the levels of these contaminants were not likely to result in noncancerous health effects. Furthermore, they reported that estimated cumulative cancer risks from air contaminants were within the EPA’s target risk zone of 1 × 10^−6^ to 1 × 10^−4^ deaths.

In 2017, the results from a public health consultation (PHC) were made public. A PHC is a written response related to a request about a specific EPA site, a location where chemicals have been released, or a location where hazardous materials are stored. PHCs can lead to many public health actions, such as removing contaminated materials, restricting site access, or requiring more health surveillance [29]. The purpose of the 2017 PHC was to assess exposure to pollutants in residential surface soil and garden produce in the Collegeville/Fairmont/Harriman Park neighborhoods. 

The 2017 PHC was requested in November 2014, when the EPA asked that the ATSDR assess soil samples from the properties of people living in the Collegeville/Fairmont/Harriman Park neighborhoods of North Birmingham. The EPA requested that the ATSDR focus its assessment on arsenic, lead, and polycyclic aromatic hydrocarbons (PAHs). Soil samples that were collected from November 2012 through January 2016, as well as garden produce samples that were collected in July 2013, were used in the analysis. Prior to remediation, the median soil concentration of arsenic was 20 ppm, the median soil concentration of lead was 157 ppm, the median concentration of a benzo(a)pyrene toxic equivalent was 0.33 ppm, and the median concentration of dibenz(ah)anthracene was 0.057 ppm [29]. In the ATSDR report, tables provided minimum, maximum, 75th percentile, 25th percentile, and mean concentrations based on different removal scenarios [29]. Based on the soil samples, the ATSDR reported in 2017 that past and current exposure to arsenic and lead could harm people’s health, especially children. As an example of health concerns, based on the laboratory data, the ATSDR concluded that approximately 5.2% of the residential properties tested had arsenic levels that were of public health concern for children who eat soil and that these children may experience or did experience acute health effects such as nausea, vomiting, and diarrhea. Approximately 2% of properties were found to have arsenic levels that could likely cause dermal problems in children. In addition, the ATSDR found that 11.8% of residential properties’ soil was at or exceeded 1 per 10,000 people, the cancer threshold for arsenic. Thus, they concluded that long-term exposure to the soil could result in excess cancer risk [29].

Furthermore, the ATSDR concluded “that long-term exposure (i.e., many years) to PAHs found in the surface soil of some residential yards increases the risk of cancer” [29]. The levels of arsenic and lead found in garden produce did not result in health concerns alone, but in combination with surface soil exposure, the report stated that there was a concern. PAHs were not found in garden produce. In addition to the federal response, the JCDH conducted a geospatial analysis for cancer, asthma, and COPD mortality in the Collegeville/Fairmont/Harriman Park neighborhoods, finding no significant difference in the 10-year rate (2000–2009) in mortality between the North Birmingham zip codes and the rest of Jefferson County, Alabama. The JCDH conducted a spatial analysis assessing adverse birth outcomes (infant mortality, stillbirths, birth defects) and found no significant differences [37]. While the JCDH found no significant differences in mortality and birth outcomes between the residents of the North Birmingham neighborhoods and Jefferson County, the JCDH results focused on mortality and not on disease burden.

## 6. Community Response

Weak enforcement of environmental regulations and an inadequate response to community complaints are concerns North Birmingham residents have continuously expressed in public notice comments and neighborhood meetings [38]. In the summer of 2013, collaborations between the EPA and residents resulted in the formation of the North Birmingham Community Coalition (NBCC), which is made up of community, business, faith, and local government representatives. The coalition was charged with developing a revitalization action plan for the neighborhoods in the North Birmingham community. The following three priorities were identified as critical to the success of any revitalization efforts in North Birmingham:

1. Environmental cleanup and restoration;
2. Pollution reduction and prevention; and
3. Multigenerational engagement and involvement.

Planning meetings were held to begin to operationalize the priorities identified, but the pattern of infrastructure development with many grassroots initiatives is only as strong as its participating entities. Poverty, racism, lack of transportation, and other forces can constrain individuals from fully participating in organizing work. The NBCC struggled with consistent leadership and consensus building in moving priorities forward in a tangible way, and as result, the NBCC organizational structure never fully took shape. Outside consultants were hired to facilitate meetings, leaving little opportunity for community members to be involved in the actual design and planning of meetings. There seemed to be a lack of a relationship between the community and the consultants. One of the main determinants of the NBCC continuing was that the consultant group did not work to identify and train community members to lead the group once they exited. 

In 2016, a subset of the NBCC group began meeting to address areas of the North Birmingham Framework Plan, which was developed in partnership with the Regional Planning Commission [38]. One major difference compared to the NBCC is that this subgroup currently actively meets with an assigned City of Birmingham senior planner to address housing, zoning, and public health issues. The original NBCC lacked that engagement. The subset group also has a committee structure led by North Birmingham residents supported by the city planner. A recent accomplishment of the group involved the downgrading of zoning categories of over 800 parcels from heavy industrial to light industrial. The residents’ continual referral to the framework plan when addressing local officials aided in that downzoning victory for North Birmingham (personal communication with City of Birmingham senior planner).

## 7. Discussion

Local and state governments have played a role in both generating the problem and offering mediocre solutions to communities impacted by polluting industries. North Birmingham’s economic landscape has always had a symbiotic relationship with heavy industry, yet healthcare facilities, businesses, and home values have declined or completely disappeared from the once thriving area. For years, pollution has impacted the North Birmingham community, causing concerns about both short- and long-term health impacts. Examining environmental justice from a public health perspective involves recognizing that communities of concern might be disproportionately affected not only because of their higher levels of exposure to environmental hazards but also because, for a variety of reasons, such exposures have a greater effect on them than on other communities. Several studies [39,40,41,42] have reviewed variations among minority populations in their susceptibilities to the effects of environmental exposures. They have reported that susceptibility such as the sickle cell trait may increase one’s susceptibility to the toxic effects of carbon monoxide or that those with diabetes may be less likely to detoxify organic solvents. Social inequality with regard to access to healthcare also serves as a susceptible factor. During multiple community meetings and public comment proceedings, North Birmingham residents have expressed concerns regarding incidents of respiratory concerns, skin issues, and cancer [35]. It is important to examine potential differences in the susceptibility of members of these communities.

Today, North Birmingham is a community still battling against environmental injustice and attempting to regain trust with government entities and other community partners.

The future of the current 35th Avenue Superfund Site being placed on the NPL is unknown. Currently, the EPA is still sampling and removing soil from contaminated properties. However, not all residents have agreed to allow the EPA onto their property, because of lack of communication, wrong messages, and time. In June 2014, Alabama’s Department of Environmental Management (ADEM) told the EPA that they were not opposed to the 35th Avenue Site being placed on the NPL if the EPA was able to reach an agreement with the potentially responsible parties for the funding of the remediation. Alabama’s share of the cleanup costs was 10%, but they informed the EPA that currently no funding source existed [43]. Five years after the site was proposed to the NPL and despite the scientific evidence deeming the site eligible for NPL status, it is still not on the list. Miscommunication about how processes work only adds to a greater lack of trust in the community.

One robust example of miscommunication and damage to the fragile trust of the community occurred in 2017. In the fall of 2017, the U.S. Court for the Northern District of Alabama filed a grand jury indictment against Joel Gilbert and Steve McKinney, two attorneys and partners with the Environmental and Natural Resources Section of the Balch & Bingham law firm, headquartered in Birmingham, and Drummond Company executive David Roberson [44]. Court documents stated that Gilbert and McKinney worked to prevent the EPA from listing the 35th Avenue Superfund Site on the NPL. The plan included advising residents of North Birmingham and public officials to oppose EPA action. Balch & Bingham paid a state representative to take official action favorable to the industry’s interests. Community residents have expressed feeling discounted and betrayed by governmental leadership.

Similar resident sentiments have been conveyed regarding the legitimacy of health department-generated data, the lack of transparency of the JCDH board of health decision-making process, perceived neglect of the North Birmingham community by the City of Birmingham, and general feelings of not being listened to by any entity with power. Many residents do not believe the health department’s findings, which were based on mortality data and not the burden of disease in the community. The community desperately wants a comprehensive health assessment conducted that focuses on diseases that are not tracked through surveillance and/or mortality data, such as neurodevelopmental and neurocognitive conditions that may be environmentally related. They want an assessment that accounts for familial history of disease, and they want a children’s health study to be conducted. These opinions are voiced at monthly meetings, but yet little has been done to respond to the community’s desire.

Despite these viewpoints, community residents, many of them members of the 1963 Children’s March, pull upon their rich civil rights history as fuel for their sustained involvement in this environmental justice battle. When EPA community organizers left, members of the community reorganized to continue their battle and join together in a meaningful way. With the help of a City of Birmingham senior planner, the community has rallied together to develop an implementation committee, which holds monthly subcommittee meetings, including a public health subcommittee meeting run by a leader in one of the six neighborhoods. Today, there are four subcommittees that focus on housing, public health, zoning, and youth development. These focus areas are what the community has identified as priorities. The subcommittee meetings are held every Monday of each month and are run by residents. Each Monday represents a different meeting of the subcommittee. All meetings are held at the local library. A senior city planner, who predominately works with the zoning subcommittee, serves as a resource for the subcommittees. 

Since 2017, these groups have conducted regular meetings to discuss pertinent items affecting the neighborhoods, and they bring in speakers and guests as the community requests. At one recent monthly meeting, the JCDH’s director attended to explain what the health department was doing to assist North Birmingham. This meeting had a large attendance, and the community had the opportunity to ask questions. The most successful aspect of these subcommittees has been that members of the community work with the city planner to move issues along to the appropriate city offices. This strategy of resident-run meetings in conjunction with working with the city has resulted in clearer lines of communication and efficiency in the flow of information back to residents.

## 8. Conclusions

The environmental justice movement has established clear goals of eliminating unequal enforcement of environmental, civil rights, and public health laws and differential exposure of some populations to harmful chemicals at home, in schools, in the neighborhood, and in the workplace. The story of North Birmingham is a complex labyrinth of social, environmental, political, and economic factors. National report findings, federal executive orders, and assessment data call for improved methodologies to mitigate the impacts of environmental pollution felt by marginalized communities, yet in North Birmingham the burden of proof continues to rest with residents to reduce and prevent further pollution in their community. Achieving environmental justice lies in the realm of equal protection and equitable distribution of resources for all communities. No one community should bear the burden of decades of heavy pollution and be forced to trade the health of their community for business. North Birmingham will continue to challenge heavy industry for a clean, safe, just, and healthy community.

## Figures and Tables

**Table 1:** Population characteristics of North Birmingham zip codes compared to Jefferson County, Alabama.

Characteristic	Jefferson County, AL	35207	35217	35234
Total population	659,460	8939	13,363	5446
% African American	42.6%	92.4%	61.2%	88.2%
Median age	37.6	35.5	40.4	49.3
% High school graduate or higher	89.4%	78.4%	80.8%	75.8%
% Unemployment	7.7%	12.1%	11.7%	17.7%
Median household income	$49,321	$23,170	$31,134	$24,228
% People whose income in the past 12 months was below poverty level	17.6%	40.6%	26.5%	34.8%
% No health insurance	10.3%	16.1%	18.1%	19.4%

**Table 2:** PM_2.5_ sampling results from the North Birmingham monitoring stations [36].

Years	Location	Annual Average Concentration (µg/m^3^)	98th Percentile of 24-h Samples (µg/m^3^)
1999–2001	North Birmingham, Monitor #1	21.6	50
	North Birmingham, Monitor #2	23.2	53
2004–2006	North Birmingham, Monitor #1	18.6	44
	North Birmingham, Monitor #2	20.4	52
2010–2012	North Birmingham, Monitor #1	13.0	27
	North Birmingham, Monitor #2	13.6	27
